# Potential threat of malaria epidemics in a low transmission area, as exemplified by São Tomé and Príncipe

**DOI:** 10.1186/1475-2875-9-264

**Published:** 2010-09-29

**Authors:** Pei-Wen Lee, Chia-Tai Liu, Virgilio E do Rosario, Bruno de Sousa, Herodes Sacramento Rampao, Men-Fang Shaio

**Affiliations:** 1 The Anti-Malaria Team of Taiwan in São Tomé and Príncipe, Democratic Republic of São Tomé and Príncipe; 2 Taiwan Urbani Foundation, Taipei, Taiwan; 3 Centro de Malaria e Doencas Tropicais/IHMT/Universidade Nova de Lisboa, Lisbon, Portugal; 4 Centro National de Endemias, São Tomé, Democratic Republic of São Tomé and Príncipe; 5 Department of Tropical Medicine, National Yang-Ming University, Taipei, Taiwan

## Abstract

**Background:**

*Plasmodium falciparum *is the major cause of malaria infection in the island of São Tomé, in the Republic of São Tomé and Príncipe (STP), with an incidence of 40 - 50% before 2004. Since 2004, through the coordination of the Ministry of Health of STP and their Centro Nacional de Endemias (CNE), an integrated malaria control programme has been intensively deployed on the island of São Tomé. Malaria morbidity and mortality decreased by 95% after three years of effective intervention. In the low transmission settings, however, malaria seasonal fluctuation can be a potential problem directly related to epidemics if ongoing control measures are interrupted. Studies on a number of associated factors with malaria epidemics and the measures taken to respond to outbreaks are presented.

**Methods:**

The integrated malaria control programme included indoor residual spraying (IRS), long-lasting insecticidal nets (LLINs), intermittent preventive therapy for pregnant women, as well as early diagnosis and prompt treatment with artemisinin-based combination therapy (ACT). Regular implementation of an island-wide IRS programme was carried out yearly in 2004-2007, and enhanced throughout the island in 2009. Malaria incidence and prevalence were estimated based on passive case detection and mass screening, respectively. Slide positivity rates were used for monitoring the beginning of a malaria epidemic or a seasonal peak.

**Results:**

A steep decline of ca. 95% of malaria morbidity and mortality was observed between 2004 and 2008 with use of the combined control methods. Malaria incidence was 2.0%, 1.5%, and 3.0% for 2007, 2008, and 2009, respectively. In April 2008, a cross-sectional country-wide surveillance showed malaria prevalence of 3.5%, of which 95% cases were asymptomatic carriers. Only 50% of asymptomatic carriers were cured with ACT treatment, while 90% of the symptomatic patients were cured by ACT treatment as confirmed with a follow up study. Malaria morbidity increased by three-fold during the first half of 2009 as compared to the same period in 2008. Over this period of six months, severe malaria was also noted in all age groups and malaria mortality increased by two-fold in children less than five years old. After an emergency IRS was deployed, with increased use of LLINs, and an active search of asymptomatic carriers was followed and given complete ACT treatment, malaria incidence decreased to less than 1% in the second half of 2009.

**Conclusion:**

At the initial stage of the integrated malaria control programme, IRS contributed to the visible effect on the rapid reduction of malaria morbidity and mortality, while this programme highlights an urgent demand for the improvement of other measures, particularly promotion of LLINs usage, with close monitoring of asymptomatic carriers and with ACT treatment in malaria transmission hotspots. In addition, both daily reports and a regular active surveillance to prevent malaria outbreaks should be established permanently, so that a fast response to epidemics can be effectively made when necessary.

## Background

The island of São Tomé, isolated in the Gulf of Guinea, is the major island in the archipelago of São Tomé and Príncipe (STP). Falciparum malaria used to be hyperendemic and predominated with a prevalence of up to 70% in some districts of STP [1]. Malaria control culminated in STP during the early 1980s using dichlorodiphenyltrichloroethane (DDT) indoor residual spraying (IRS) and weekly prophylaxis with chloroquine, which rapidly resulted in reducing the malaria prevalence to less than 1%, and incidence, for at least one year, fell to zero [2]. However, the programme was disrupted in 1982, due to financial difficulties and lack of a long term control planning [2]. Consequently, and following a severe epidemic in 1986, malaria rebounded and caused high mortality in small children [2]. The situation deteriorated with the emergence of mosquito resistance to DDT and the appearance of *Plasmodium falciparum *chloroquine-resistant strains. Since then, malaria became a major public health problem until 2003, when the Taiwan International Cooperation and Development Fund (ICDF) collaborated with the São Tomean Government to implement a nationwide indoor residual spraying programme to reduce malaria transmission by *Anopheles *mosquitoes [3]. Of the two islands of STP, Príncipe was selected as the pilot place for IRS in 2003 [3]. Subsequently, the nationwide scaling-up of preventive strategies included yearly cycle of IRS with alphacypermethrin starting in 2004, followed by intermittent preventive therapy (IPT) in pregnant women with sulphadoxine-pyrimethamine (SP) in 2004, the widespread use of artemisinin-based combination therapy (ACT) in 2005, and a national campaign of distribution of long-lasting insecticidal nets (LLINs) also in 2005 [4]. The remarkable reduction in malaria morbidity and mortality encourages serious consideration of eliminating malaria on these islands [4-6]. Although rapid control of malaria by means of IRS of alphacypermethrin was noted in STP [3], its sustainability can be a challenge even current malaria control strategies are better than those applied in the 1980s. At the present time, the use of alphacypermethrin in IRS is still fully effective and ACT resistance has not been observed. However, dramatic decreases in morbidity and mortality may be short-lived because both social problems and political disputes frequently hinder the progress of integrated control programme in the island. Currently, malaria is maintained as asymptomatic and sub-patent infections, which highlight the alarming prospect of malaria to become a serious public health problem in STP. The previous 1980 failure in STP on attempts for malaria elimination, which was followed by major mortality, can be repeated in the future if sustainability of the programme is not maintained [2]. To avert a possible large-scale epidemic in a low and unstable malaria transmission setting, investigation into those insufficiencies and establishment of all possible means were carried out to consolidate the achievements of the current integrated programme.

## Methods

### Sites

The island of São Tomé with a total area of 860 km^2 ^is situated in the Gulf of Guinea, 400 kilometers off the coast of West Africa. This volcanic island has a hot and humid equatorial climate, which is characterized by the alternation of two distinct seasons: the dry and temperate season, from June to August, and the hot and rainy season, from September to next May. In 2008, some 160,000 inhabitants were living on the island of São Tomé. The population density (Additional file 1) is highest at Agua Grande district and lowest at Caue district [7]. Most houses were wooden built with zinc-plated roofs but the island also presents cement and modernized construction.

The programme was initiated in 2004, and a molecular diagnostic laboratory was set in the main island of São Tomé, following STP government directives for malaria control and for ethical clearance throughout the implementation of the programme. Informed verbal consent was obtained from residents who answered a short questionnaire, which included information on the use of bed nets. Parents responded on behalf of infants and children.

### Malaria control strategies

#### IRS

Under the full support by Taiwan International Cooperation and Development Fund, regular implementation of a nationwide IRS program was carried out yearly for consecutive three years on the island of São Tomé from 2005 [3]. Regular IRS was not carried out simultaneously through the country but district by district, from south to north. It took about 11 months to complete a yearly cycle. The last regular cycle of IRS was completed at Lobata in March 2007 and at Agua Grande in November 2007. Visiting households and checking for mosquito mortality by cone bioassays was carried out regularly and labeling of houses under IRS treatment was also carried out. To evaluate residual activity of alphacypermethrin, standard cone bioassays of the sensitivity of Anopheles to the insecticides were undertaken bimonthly according to WHO protocols [8]. This programme was complemented with LLIN, in 2005, and larviciding in 2007.

In addition, emergency IRS was applied in a focalized manner by targeting community at risk for malaria epidemics. Emergency IRS was carried out to respond to a malaria outbreak at Lobata in June 2008 and then widened to cover the whole district. Emergency IRS also targeted malaria transmission hotspots at Agua Grande in November 2008, extended to the whole district in March 2009, and continued to cover other neighbour districts afterwards.

#### LLINs

Since 2005, 79,000 LLINs have been distributed freely to children and pregnant women with funding from many international donors including the Global Fund against AIDS, Tuberculosis and Malaria (GFATM). An LLINs ownership rate of two nets per household was >80% [9]. Over the periods 2007-2009, the usage of LLINs in both age groups was investigated (results obtained by using questionnaire when IRS and active surveillance were carried out).

#### Larviciding

Larviciding operation by use of *Bacillus thuringiensis israelensis *(Bti, VectoBac G, Lot number 145-077-N8, 200 ITU/mg, Valent Bioscience Corporation, Libertyville, Ill), was regularly applied (once per week) to breeding sites through the whole year. In São Tomé Island, more than 50 permanent breeding sites distributed in six districts were identified and these stayed active during the dry season. Temporary breeding sites appeared during the rainy season were also examined and treated. Quantification of larvae in the breeding sites and the percentage reduction in larval mosquito densities was performed as previously described [6].

#### IPT

Over the periods 2005-2009, about 6,000 women were pregnant each year. Under the supervision by clinicians or nurses, pregnant women received sulphadoxine and pyrimethamine (SP) for IPT during the 4^th ^and 7^th ^month of pregnancy [10]. Coverage steadily increased from 60% in 2005 to 85% in 2009 [11].

#### ACT

With support from Global Fund and ICDF Taiwan, artesunate-amodiaquine has become the first-line treatment for uncomplicated malaria since 2005. Artemether-lumefantrine (Coartem^®^, Novartis) is currently used as the second-line drug for malaria treatment. Uncomplicated malaria patients diagnosed by passive case detection or active case detection received ACT treatment or were admitted to hospital for quinine treatment when showing severe symptoms. Women suffering from malaria during their first trimester of pregnancy were also treated with quinine. Since 2006, over 90% of uncomplicated symptomatic malaria patients received ACT treatment [12].

### Data collection procedures

#### Diagnosis

Malaria is diagnosed through passive case detection by using optical microcopy in hospitals and the district health centers and through mass screening by use of the rapid diagnostic tests (RDTs, ICT Diagnostics). To verify both the sensitivity and specificity of RDTs, in 2005, 924 cases of fever (body temperature >37.5°C) were tested by both RDT and blood film examination. The discrepancy in results from these two methods was further clarified by polymerase chain reaction (PCR) [1,13], carried out locally. Malaria positive cases were defined as either microscopic positive or/and PCR positive.

#### Passive case detection

Patients were suspected clinically to have malaria and received blood film examination at health stations. Microscopic reading on blood films was performed according to CNE diagnostic protocols. Throughout the control programme during 2003-2009, microscopic examination has been used as the gold standard method as previously described [6]. For each district health center, at least two trained microscopists examined the blood films simultaneously and a third one clarified any discrepancy in results. A blood film was declared negative when no parasite was detected in 200 fields. Once malaria patients were diagnosed by passive case detection, within one week, members of the patient family in the same house and residents in adjacent houses were visited and asked by a mobile team (consisting of a nurse and a technician) to supply a blood sample for microscopy examination.

#### Mass screening

Malaria mass screening began with a cross-sectional country-wide survey, wherein all residents were submitted to RDT. Between 2 April and 2 May 2008, 132,934 people participated which accounted for 90% of the total population on the island of Sao Tome. In addition, regional malaria surveys by mass screening were also conducted both at Lobatar and Agua Grande districts in April 2009. Internal quality control of RDTs included an immediate blind second reading of 100% of the RDTs. In case of disagreement, two technicians re-examined the RDT together and decided on the reading. All positive cases found by RDTs were examined by optical microscopy of Giemsa-stained blood smears. The technician recording the microscopic result was unaware of the corresponding RDT results. Final results were verified by PCR if inconsistent findings were observed between RDT and blood films.

### Study methods

#### Follow-up, evaluation, and monitoring

Patients found positive for malaria infection, either by passive case detection or by active detection (mass screening), were given anti-malaria treatment according to CNE guidelines. A registration card was filled in with the patient's name, sex, age, weight, body temperature, parasitaemia, drug regimen, and address (village or locality) was kept for follow-up. An uncomplicated malaria case was defined with a blood count of *P. falciparum *asexual parasitaemia >0 parasites/μl, and not fulfilling the criteria for severe malaria as previously described [6]. The uncomplicated malaria cases treated with ACT at home, were followed up by a mobile team (consisting of a nurse and a technician), which actively visited patients by taking blood films for microscopic examination two weeks after the three-day treatment. Cure rate was calculated as the percentage of cases having negative results by blood film examination two weeks after a three-day ACT treatment.

#### Identifying epidemic malaria

Several epidemic threshold methods have been developed [14]. However, there is no absolute standard. To reduce the number of malaria epidemics and the number of cases during epidemics, a daily malaria report system has been established and the method of constant case count thresholds is used [14]. For comparison, the weekly trends of slide positivity rate (SPR) and monthly trends of incidence, in 2007, were used as baselines. The daily records of malaria detection in health stations were collected and reviewed, and analysed in order to calculate SPR weekly at each district health center as previously reported [15]. A report of the *P. falciparum *incidence was made monthly at CNE. A level of 5% for SPR was set up as a threshold to evaluate the control programme. An increase of weekly SPR above 5% induced an active detection response, including examination of the patient's family and neighbors, by using RDTs. A double increase of malaria new cases in one week as compared to the previous week was regarded as the possible occurrence of an early epidemic sign. A double increase in malaria morbidity, as compared to the same month of previous year, was treated as an epidemic.

#### Response to malaria epidemics

Emergency IRS was intensively carried out within one week, with an area of 1 km semi-diameter from the center of cluster infection. Emergency IRS was enhanced and enlarged to the whole district and neighbor districts if necessary. The usage of LLINs was promoted through community health education and medium propaganda. All positive cases for malaria were received ACT treatment and followed up.

### Data analysis

Statistical analyses were performed using SPSS version 17.0. The annual trends of morbidity for the classes "<5 years old", "≧5 years old", and "pregnancy", were tested by Poisson regression for the years 2003 through 2009. The association of IRS and LLINs on malaria block transmission (refers to vector control, whatever the method) was measured through logistic regression analysis. Dependent variable is malaria (positive or negative). Independent variables are types of protection (unprotected, LLINs, IRS + LLINs and IRS). The reference class used was "IRS". No confounding variables were available to be used in the model. In the Negative Binomial models, dependent variable is malaria counts while independent variables are year (2003 to 2009, reference class 2003) and age class (<5 years, >= 5 years and pregnant, reference class <5 years).

## Results

### Effect of vector controls

The average acceptance rates of IRS for dwellings and outhouses throughout this island were 87%, 83% and 75% for 2005, 2006 and 2007, respectively. The third regular IRS (2007) had a lower coverage (<80%), particularly observed at densely populated districts in and around the capital city (Agua Grande, Me-Zoxi, and Lobata). (Additional file 1).

Over the periods 2005-2007, the usage of LLINs in both age groups were constantly low than 50% (results obtained by using questionnaire when IRS and active surveillance were carried out). The usage rate of LLINs increased over 50% (Table 1) due to the alert of possible malaria epidemics after an outbreak at Agua Grande district occurred in Nov 2008.

**Table 1:** Effect of combining indoor residual spraying (IRS) and long-lasting insecticide-treated nets (LLINs) interventions on malaria infection in São Tomé during the first half year of 2009

Intervention	Malaria cases	Total cases	OR	95% CI	P value
IRS only	492	3775	1		<0.001
Unprotected	355	1605	1.895	1.629 - 2.204	<0.001
LLINs only	136	1934	0.505	0.414 - 0.615	<0.001
IRS + LLINs	95	5149	0.125	0.100 - 0.157	<0.001

IRS: indoor residual spraying; LLINs: long lasting insecticide-treated nets;

OR: odds ratio; IC: confidence interval.

An average mosquito mortality of 80% is considered an effective residual killing by cone bioassay, which showed that the alphacypermethrin steadily maintained its residual efficacy for one year when it was applied to wooden walls but its insecticidal effect only persisted for six months when applied to cement walls. This long-lasting residual effect of alphacypermethrin has been constantly observed since 2005.

Larviciding assay showed that 100% mortality of young larvae was achieved after 24-hr Bti exposure at all permanent breeding sites during the dry season, but varied greatly from 20% to 90% during the rainy season.

In May - Jun 2009, 12,463 inhabitants living at malaria transmission hotspots (located at Agua Grande and Me-Zoxi) were examined by active detection and interviewed if they lived in IRS-treated dwellings and/or slept under LLINs during the first half year of 2009. Table 1 shows that unprotected residents were 1,605 (of which 355 had malaria), 3,775 residents were under IRS programme (of which 492 had malaria), 1,934 under LLINs (of which 136 had malaria), and 5,149 under both IRS and LLINs (of which 95 had malaria). The results of the logistic regression analysis in Table 1 show that the combined use of IRS and LLINs has an additional protective effect against malaria when compared to the use of IRS alone (OR = 0.125, 95% CI: 0.100 - 0.157, p < 0.001). Being unprotected increases the odds by 2 (OR = 1.895, 95% CI: 1.629 - 2.204, p < 0.001) of that for IRS protection alone, while using LLINs alone, when compared with IRS protection alone, decreased the odds by almost a half (OR = 0.505, 95% CI: 0.414 - 0.615, p < 0.001). This is a significant finding since the number of people slept under LLINs alone is only 16% (1,934) of the interviewees, when compared with the total of 12,463 people in the study, of which 72% (8,924) of people lived in IRS-treated houses. It was also noted that the usage of LLINs reached 57% (7,083) whether a person lived in an IRS-treated house or not. It seems that the only factor increasing the odds of being infected with malaria is being unprotected (OR = 1.895, Table 1). Both the combined use of IRS and LLIN and LLIN alone have additional protective effect against malaria when compared to the use of IRS alone.

### Malaria morbidity

**Figure 1:**
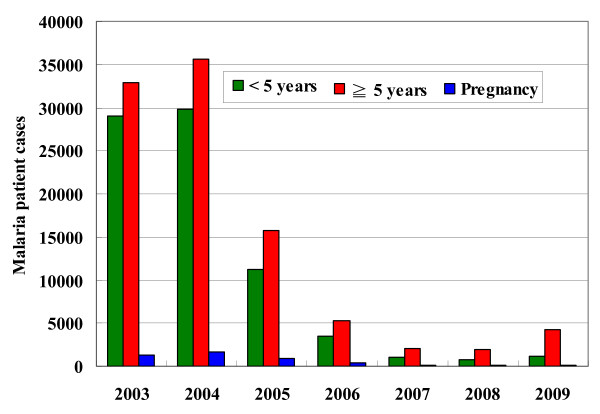
**Malaria morbidity on the island of São Tomé 2003-2009**.

**Figure 2:**
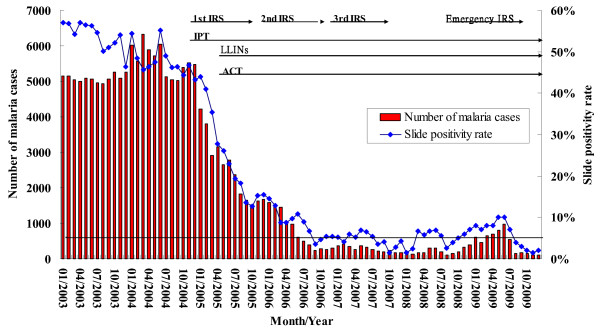
**Monthly trends of malaria morbidity and slide positivity rate (SPR) and malaria cases on the island of São Tomé 2003-2009**.

The annual number of malaria cases exceeded 60,000 before 2005, with a high incidence of over 400 per 1,000 population (Figure 1). A steep decline by 95% of malaria morbidity was noted between 2004 and 2007. In 2008, malaria incidence was less than 20 cases per 1,000 population at risk. Malaria incidence kept at a low level but varied from district to district, with 2.3% at Lobata and 0.7% at Caue (Additional file 1). However, a three-fold increase of malaria morbidity was found in the first half of 2009 when compared to the same period of 2008 (3827 cases vs. 1244 cases, P < 0.05, Figure 2). On the other hand, over the second half of 2009, malaria incidence reduced to less than 1% (1645 cases found in the second half of 2009). Nevertheless, throughout the whole year in 2009, malaria incidence increased notably nationwide except at Lemba and Caue districts (Additional file 1).

Number of pregnant women with malaria decreased by 95%, from its peak, 1692 cases in 2004 to 81 cases in 2009 (Figure 1). Malaria incidence in pregnant women also declined steeply, from 30.8% (1692/5486) in 2004, 1.6% (99/6112) in 2007, 1.5% (95/6198) in 2008, and to 1.3% (81/6253) in 2009. There has been no malaria death in pregnant women since 2006.

Due to the problem of over-dispersion (a necessary condition to apply Poisson Regression), application of a Negative Binomial Regression model allows for the presence of overdispersion. In this model the reference classes are the year 2003 and children of less than five years of age for the main effects "Year" and "AgeClass", respectively.

Table 2 shows that not all the parameters are significant in the model (p > 0.05). As the year increases the coefficient becomes more and more negative, with the exception of year 2008 and 2009. There are no statistically significant differences for the years 2003, 2004 and 2005, but after that the coefficients suggest that fewer and fewer cases of malaria infections are observed. The model suggests that the 2009 would have a lower impact in the cases of malaria than the year 2008.

**Table 2:** Parameter (malaria morbidity) estimates by Negative Binomial regression analysis

Parameter	Coefficient	Standard error	95% Wald CI	P value
(Intercept)	9.984	0.6329	8.743 - 11.224	<0.001
Year 2009	-2.805	0.8311	-4.434 - -1.177	0.001
Year 2008	-3.052	0.8229	-4.665 - -1.439	<0.001
Year 2007	-2.886	0.8205	-4.494 - -1.278	<0.001
Year 2006	-1.759	0.8205	-3.367 - -0.151	0.032
Year 2005	-0.687	0.8188	-2.291 - 0.918	0.402
Year 2004	0.116	0.8169	-1.485 - 1.717	0.887
Year 2003	0 ^**a**^			
Pregnancy	-2.621	0.5412	-3.682 - -1.561	<0.001
≧5 years old	0.583	0.5449	-0.485 - 1.651	0.284
<5 years old	0 ^**a**^			
(Scale)	1 ^**b**^			

Dependent Variable: malaria counts

Model: (intercept), Year, AgeClass

^**a **^Set to zero because this parameter is redundant.

^**b **^Fixed at the displayed value.

Relatively to the AgeClass factor, when compared to children of less than five years of age, being five years of age or older does not contribute to an increase of malaria cases (p = 0.284), while being pregnant contributes to lower cases of malaria infection.

### SPR

SPR of malaria episodes was 50% over the period 2003-2004 but a steep decline of SPR has been observed since with averages of 25% in 2005, 9% in 2006, 5% in 2007, and 4% in 2008 (Figure 2). When districts were taken into account separately in 2008, the highest annual SPR of 5.2% was observed at Lobata, followed by 4.6% at Agua Grande. At Lobata, SPR abruptly increased to 12% in May and then steadily declined to below 5% in October 2008 and thereafter. Increased SPR over 5% was also noted at Agua Grande in November, followed by Me-Zoxi in December 2008 and Cantagalo in January 2009. Overall, monthly SPR increased over 5% in November 2008 and steadily approached to 10% during the first half of 2009 but declined below 5% after July 2009 (Figure 2).

### Malaria prevalence

In April 2008, a nation-wide mass screening was carried out in this island which showed malaria prevalence of 3.5% (4631/132934). *P. falciparum *is the major species responsible for 90% of malaria infections. Malaria prevalence varied in different districts, being the highest at Lobata (4.1%), followed by Me-Zoxi (3.7%), Agua Grande (3.5%), Cantagalo (3.2%), Lemba (2.0%), and the lowest at Caue (1.9%) (Additional file 1). Among the positive cases, 4417 cases (95%) were asymptomatic and 214 (5%) cases were symptomatic. There were 536 cases (12%) of children under 5 years old and 4086 cases (88%) above that age. Of the 9 pregnant women with malaria, 7 (78%) were asymptomatic.

Active surveillance implemented at some malaria hot spots in April 2009, showed malaria prevalent rates of 4.3% (1593/37055) at Agua Grande and 4.1% (391/9531) at Me-Zoxi, 4.2% (243/5783) at Lobata, and 3.5% (43/1231) at Cantagalo (Additional file 1). Malaria prevalence was slightly increased during the recent seasonal epidemic suggests that it is not a sensitive indicator for monitoring malaria epidemics.

### ACT treatment and cure rate

Among 4,417 asymptomatic malaria carriers found by mass screening in April 2008, 2932 cases (66%) were followed up. Among 3,827 patients with symptomatic malaria confirmed by passive detection during the period of Jan - Jun 2009, 3,479 cases (91%) completed the follow-up.

Cure rate for symptomatic malaria patients with ACT treatment ranged from 86% to 96% in different districts, with an average of 90% (3,131/3,479, Additional file 1). However, only a half (1,455/2,932, 50%) of asymptomatic malaria carriers cured with ACT treatment. Cure rate for asymptomatic cases varied greatly from 35% at Me-Zoxi district to 95% at Caue district. Among the cases with ACT treatment failure, incomplete treatment (not taking full course of ACT treatment) was noted in both symptomatic and asymptomatic cases, with 90% (314/348) and 95% (1,405/1,477), respectively. A minority completed ACT treatment but was not cured, i.e., 1.0% (34/3,479) and 2.5% (72/2,932) for symptomatic and asymptomatic cases, respectively.

### Malaria epidemics

**Figure 3:**
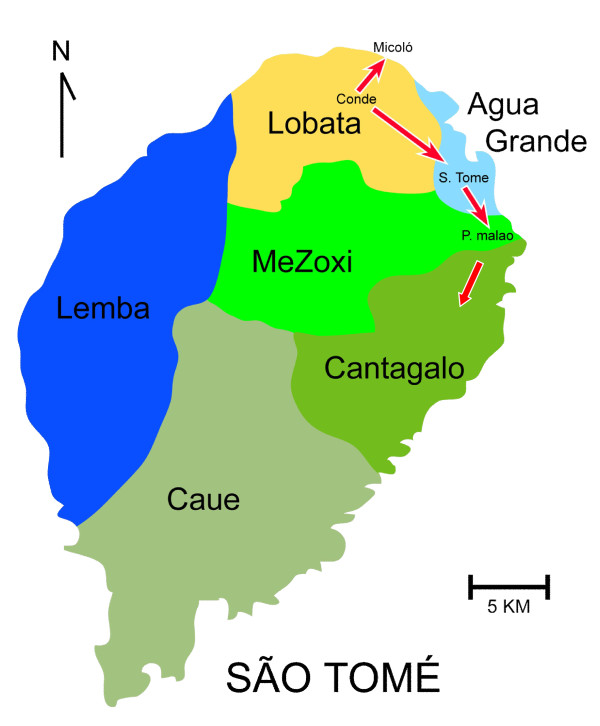
**Map for malaria epidemics on the island of São Tomé 2008-2009**.

In mid-May 2008, an increase of malaria cases was first observed at Lobata. Malaria morbidity increased by 170% (182 cases vs. 68 cases) in May 2008 when compared to the same period in 2007. An increase of SPR over 10% at Lobata in May provided an indicator for malaria resurgence. The outbreak was under control within two months after emergency IRS was applied to the hot spot areas within 2 Km diameter. Subsequently, malaria epidemics occurred at Agua Grande in November 2008, when malaria cases increased by 3.5 folds (203 cases vs. 45 cases) when compared to the same period in 2007. The operation of emergency IRS at Agua Grande, however, failed to reduce the transmission until July 2009. The epidemics started from Lobata in May, followed by Agua Grande in Nov, and Me-Zoxi in Dec 2008, and then spread to Cantagalo in Jan 2009 (Figure 3). Emergency IRS was enlarged throughout Agua Grande/Me-Zoxi/Cantagalo districts and mass screening for malaria at these districts were also intensively carried out simultaneously. Eventually, the epidemics were fully under controlled in July 2009. During the second half of 2009, SPR kept at a level of <5% and malaria incidence was less than 1%.

### Severe malaria and case-fatality rates

**Figure 4:**
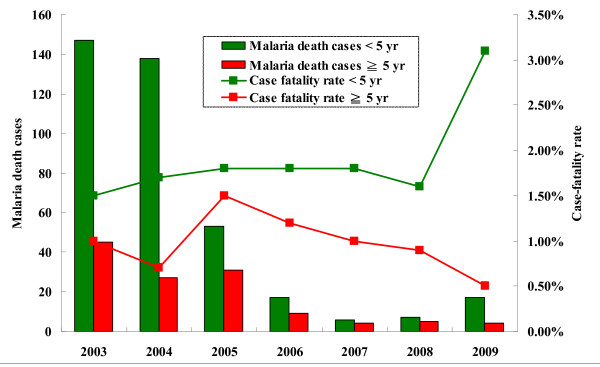
**Severe malaria and case fatality rate on the island of São Tomé 2003-2009**.

Malaria mortality was high before 2005 with 200 deaths recorded annually (Figure 4). After 2005, Malaria deaths decreased remarkably, 10 in 2007 and 12 in 2008, but surged to 17 cases in the first half of 2009 and then declined to four cases in the second half of 2009 (Figure 4).

The occurrence of malaria epidemic at Agua Grande from the end of 2008 to the first half of 2009 reflected on a notable increase of severe malaria cases (hospitalized patients) at the corresponding months. Severe malaria increased by 130% in the first half of 2009 when compared with that at the same period of 2008 (1,180 cases vs. 514 cases, P < 0.05). The increase of severe malaria cases was found for both age groups but not in pregnant women. Severe malaria in the age groups over 5 years old increased by 145% (672 cases vs. 274 cases, P < 0.05) and children under five years increased by 110% (508 cases vs. 240 cases, P < 0.05). Since 2007, 43 malaria deaths have been reported, 30 (70%) deaths were children under 5 years old (Figure 4). Overall, case-fatality rates in severe malaria patients did not vary significantly during the period 2007-2009, i.e., 1.4% (10/728) in 2007, 1.2% (12/991) in 2008, and 1.5% (21/1,383) in 2009.

However, case-fatality rates in children less than five years almost doubled in 2009, i.e., 1.8% (6/335) in 2007, 1.6% (7/436) in 2008, and 3.1% (17/546) in 2009. On the other hand, case-fatality rates in severe malaria patients with age over 5 years old declined from 1.0% (4/393) in 2007, 0.9% (5/555) in 2008 to 0.5% (4/837) in 2009.

As in the previous analysis, a similar behaviour for the factor Year related to severe malaria is noted (Table 3). As the year increases the coefficient becomes more and more negative, with the exception of years 2007 until 2009. There are no statistically significant differences for the years 2003 and 2004 (p = 0.869), but after that the coefficients suggest that fewer and fewer cases of severe malaria are observed when compared to the reference class 2003. The model suggests that there is a slight variation of the impact after 2007, decreasing in 2008 and 2009.

**Table 3:** Parameter (severe malaria) estimates by Negative Binomial regression analysis

Parameter	Coefficient	Standard error	95% Wald CI	P value
(Intercept)	8.927	0.7322	7.492 - 10.362	<0.001
Year 2009	-2.518	1.023	-4.823 - -0.813	0.006
Year 2008	-2.664	1.009	-4.642 - -0.685	0.008
Year 2007	-2.974	1.008	-4.950 - -0.998	0.003
Year 2006	-2.124	1.003	-4.090 - -0.171	0.034
Year 2005	-1.038	1.001	-3.001 - 0.925	0.300
Year 2004	-0.165	1.000	-2.125 - 1.795	0.869
Year 2003	0 ^**a**^			
≧5 years old	-0.101	0.5529	-1.185 - 0.983	0.855
<5 years old	0 ^**a**^			
(Scale)	1 ^**b**^			

Dependent Variable: malaria counts

Model: (intercept), Year, AgeClass

^**a **^Set to zero because this parameter is redundant.

^**b **^Computed based on the Pearson chi-square.

The AgeClass factor is no longer a statistical significant factor in the model (p = 0.855). No problem with overdispersion in this case. In this model the reference classes are the year 2003 and children of less than 5 years of age for the main effects "Year" and "AgeClass", respectively.

Table 4 shows that fewer and fewer cases of malaria mortality are observed after 2004 when compared with the year 2003. Nevertheless, there is a decrease of this impact after the year 2007. Relatively to the AgeClass factor, when compared to children of less than 5 years of age, both being 5 years of age or older and being pregnant contribute to lower cases of malaria mortality with a stronger effect on the later case.

**Table 4:** Parameter (malaria mortality) estimates by Poisson regression analysis

Parameter	Coefficient	Standard error	95% Wald CI	P value
(Intercept)	4.965	0.0990	4.771 - 5.159	<0.001
Year 2009	-2.318	0. 3112	-2.928 - -1.708	<0.001
Year 2008	-2.778	0.3850	-3.532 - -2.023	<0.001
Year 2007	-2.960	0.4197	-3.783 - -2.137	<0.001
Year 2006	-2.005	0.2704	-2.535 - -1.475	<0.001
Year 2005	-0.820	0.1685	-1.150- -0.490	<0.001
Year 2004	-0.139	0.1366	-0.406 - 0.129	0.310
Year 2003	0 ^**a**^			
Pregnancy	-4.333	0.5826	-5.475 - -3.192	<0.001
≧5 years old	-1.099	0.1326	-1.359 - -0.839	<0.001
<5 years old	0 ^**a**^			
(Scale)	1.675 ^**b**^			

Dependent Variable: malaria counts

Model: (intercept), Year, AgeClass

^**a **^Set to zero because this parameter is redundant.

^**b **^Computed based on the Pearson chi-square.

## Discussion

A five-year integrated malaria control programme has been intensively carried out on the island of São Tomé, since 2004, and produced a remarkable decline in malaria morbidity and mortality. However, both malaria morbidity and mortality increased in the end of 2008, one year after the last (3^rd^) country-wide IRS which ended in 2007, despite the persistent implementation of other three measures. In the low transmission settings, malaria seasonal epidemics occurred in the middle of 2008 and in the first half of 2009 when severe malaria increased in all age groups and case-fatality rate increased in young children. By prompt response and effective management, action has been taken to avoid a return of devastating malaria epidemic.

The same integrated malaria control programme has been applied to both Príncipe and São Tomé Islands since 2003, and 2004, respectively. Current status of malaria in Príncipe is low and stable [6] while in São Tomé is low but unstable. The difference in results between these two islands after a five-year integrated malaria control programme could be associated with the following points:

a) malaria prevalence was relatively low (1.1% in 2006) in Príncipe while was relatively high (3.5% in 2008) in São Tomé after the 3-year IRS programme was intensively carried out. It was incapable of dealing with treatment and follow-up for near five thousand malaria cases simultaneously in São Tomé, due to insufficiency of manpower and facility. Both follow-up and cure rate for asymptomatic malaria carriers were low (only two thirds of asymptomatic cases completed the follow-up, of which less than a half cured with ACT treatment) in São Tomé but these were achieved with success in Príncipe (all asymptomatic cases were followed up and ACT treatment cure rate was >90%). The failure of ACT treatment in São Tomé was mainly due to non-compliance.

b) coverage of IRS has been observed constantly high in Príncipe but decreased by year in São Tomé throughout the three-year IRS programme. Less than 80% of households accepted 3^rd ^IRS in São Tomé in 2007, with an even lower coverage (<70%) found at some fishing villages in the north-east coastal region, the more densely populated area. In Príncipe, the high coverage of IRS (90%) integrated with three other effective control strategies has made malaria low and stable for three years without additional IRS. In São Tomé, however, the low coverage of the 3^rd ^IRS could not produce a longer lasting effect on malaria blocking transmission and this was worsened by the low usage of LLINs and insufficient management of asymptomatic carriers.

c) a warning system by early reports and prompt response to malaria outbreaks has been successfully operated in Príncipe but not fully effective in São Tomé due to the incapability and poor performance in population dense districts.

It is not easy to say with certainly whether or not a particular control measure has had any specific impact on malaria as the effects of different interventions are not easily separable. Although IRS contributed strongly to the visible effect on the reduction of malaria morbidity and mortality at the early phase of integrated control programme in São Tomé [3], the role of LLINs, implemented at later stage, with IRS, had an additional protective effect to block malaria transmission when compared to the use of IRS alone. LLINs synergistic effect while associated to IRS on the blocking of malaria transmission has been reported elsewhere [16]. This is contrast to the finding in the Príncipe programme [6], which showed no incremental benefit associated with use of LLINs in low malaria transmission settings that had been IRS treated with high coverage. Results from São Tomé programme indicated that LLINs had a synergistic effect with IRS on the blocking of malaria transmission when IRS coverage rate (72%) was not satisfied, while this synergistic effect can be seen when an increase in the usage of LLINs (57%) was noted.

Annual rainfall did not vary significantly on the island over the years 2003 - 2009 [17]. In 2008, among the six districts, rainfall at the south district (Caue) was 4-fold as much as that at the north district (Agua Grande). However, Caue had the highest IRS coverage (>90%) in 2007, the lowest malaria incidence in 2008, and no epidemics occurred. Therefore, rainfall cannot be blamed for the occurrence of malaria epidemics. It has been reported that rainfall cannot be incriminated as a major cause for malaria changes, which are mainly due to the combination of climatic, human, and operational factors [18]. An insufficient coverage of IRS (<80%) in 2007 and the delay of IRS operation in 2008 could be associated with the occurrence of epidemics in the middle of 2008 at Lobata and later on (from the end of 2008 to the first half of 2009) at Agua Grande/Me-Zoxi/Cantagalo districts. In addition, the time sequence of malaria outbreaks followed the geographic pathways (main roads) suggests that population migration play a role in the occurrence and spread of malaria epidemics.

The persistent residual effect of alphacypermethrin on wood walls by IRS has been reported to last for one year [3], but the duration of protective effect by IRS may be affected by the degree of coverage. Two south districts, Lemba and Caue with higher coverage of IRS (85-90%) did not have any epidemic even two years had passed since the last cycle of IRS in 2007, while other districts with low coverage (70-75%) had epidemics one year after the last cycle of IRS in 2007, despite three other control measures were sustained. Higher coverage rate of IRS on the island of Príncipe has ensured to reduce malaria morbidity low enough to be managed and followed up completely.

An emergency IRS programme successfully rescued Lobata from a malaria epidemic as such operation was completed before the rainy season and reached a satisfying coverage (>80%) within one week. However, failure to control the epidemic at Agua Grande in a similar situation was due to two reasons: the IRS operation was carried out during the raining season and there was low coverage of this densely populated area surrounding the capital city.

The residual period of the synthetic insecticides can be affected by different wall surface types which as observed elsewhere [19]. The duration of a residual effect of alphacypermethrin on cement walls is far shorter than that on wood walls [3]. In the urban districts where epidemics occurred, cement houses were commonly seen, while in rural districts wooden houses predominate and here epidemics did not occur. The difference in the type of housing (wall surface type) is a factor to take into account in a programme which includes IRS as well as its calendarization. Operation of the fourth yearly national-wide IRS should be initiated in early 2008 according to an original planning by a local non-government organization supported by Global Fund but was far behind schedule. The delay has interrupted regular IRS programme for more than 18 months which could be one of the causes for these malaria epidemics. Many international parties participated in the malaria control in this country but poor coordination (STP cabinet reshuffled three times in 2008) has been a serious problem. Only if the resources from multiple international parties are well integrated by the STP government, a successful programme can be secured.

The poor acceptance of IRS in some districts on the island of São Tomé can be attributed to several reasons, mainly including skin allergic reaction to alphacypermethrin [20], little impact on the control of *Culex quinquefasciatus *(the main cause of mosquito biting nuisance in urban areas) [21], and believing malaria being no more a serious disease [6]. Other causes of refusal of accepting IRS were bad smell of the insecticides, poisoning of domestic animals, poisoning of children, and that insecticides may cause infertility to family members which have corroborated another report from Tanzania [22]. Some socioeconomic problems such as being afraid that dwellings were searched for the stealing of electricity, and shame due to lack of furniture indoors also caused the refusal of IRS. On the other hand, higher refusal rate of IRS was also noted from better households because their owners, socially much better off, assumed that their cement houses were safer than wooden houses, associated to poverty, and kept mosquitoes away. Legislation to reinforce spraying is highly needed as refusal of spraying will have major consequences in the future.

Although a previous report showed SP had 20% clinical failure and high frequencies of resistance-associated mutations [23], results from our recent study indicated that the frequency of quintuple mutations (*dhfr *triple + *dhps *double) in STP was steady around 10% from 2005 to 2008, which may be due to the removal of drug pressure by the change of treatment policy from SP to ACT in 2005. Neither malaria morbidity nor severe malaria increased in pregnant women during the recent epidemics. Moreover, no malaria mortality has been reported in pregnant women since 2006. The high coverage of IPT with other control methods may have contributed to this result.

In addition to those difficulties encountered in Príncipe [6], several other problems have arisen, which require urgent reviewing of the control methods used in São Tomé:

a) both the low successful rate for follow-up and the incomplete ACT treatment for the asymptomatic carriers indicate evaluation and monitoring need to be enhanced greatly. ACT has been extensively applied to uncomplicated malaria in this country for more than four years. However, full compliance of patients to ACT treatment cannot be warranted in a context of routine use of ACT, particularly for asymptomatic carriers who have no intention to complete the treatment. Additionally, under-dosing is quite a common practice in many households because of poverty and the fact the clinical cure of fever is what matters to many individuals [24]. To overcome the potential problem of poor adherence to drug regimens, particularly for asymptomatic carriers and small children, the deployment of direct observation and treatment through the community by specially-assigned nurses should be applied.

b) a double increase of case-fatality rate in children less than 5 years old during recent epidemics indicates that capabilities of the referral system and hospital case management for young children need to be enhanced imminent. The challenges currently facing malaria control in this country are poor quality of health care, poor governance, inadequate human resource (particularly short of paediatricians), insufficient management of capacity and unaffordable maintenance [25]. The existing scenarios of prevailing poverty, week infrastructure, poor coordination, and a lack of motivation of health workers, are all challenges to be confronted [26].

c) an increase in the tendency of Anopheles mosquitoes to feed outdoors and/or earlier in the evening has been discussed. The main vector in Sao Tome is *Anopheles gambiae s.s*, a highly anthropophilic sylvatic species, whose exophagy and exophily may limit the impact of interventions such as IRS and LLINs [3,27]. Recent surveys using night bite captures revealed that biting by *An. gambiae *is six-fold higher outdoors than indoors around the capital city. A change in vector biting behaviour by IRS was previously documented in Solomon islands [28]. A favourable scenario for malaria transmission is that local people increase outdoor activities at night, such as watching TV in public open places, which greatly enhances the exposure, mainly in older people to mosquito bites. Further investigation into behavioural changes is necessary.

The current low malaria infection is mainly maintained through asymptomatic carriers. Protective immunity in communities may wane and local residents are therefore vulnerable to explosive epidemics that can increase severe malaria and cause high case fatality rates [29,30]. Considering that the environment did not change dramatically and that the annual rainfall did not vary considerably while this programme was carried out, the increase of severe malaria morbidity but not mortality was noted in old age group during the recent seasonal epidemics may reflect some changes in the status of acquired immunity but not lost immunity.

Despite various successes in malaria control on several countries including some tropical islands, there is no evidence yet to show that malaria elimination can be achieved and maintained in areas where malaria transmission is currently high (one or more malaria cases per 1,000 inhabitants per year). Initial evidence from STP, Zambia, the islands of Zanzibar (United Republic of Tanzania) and the island of Bioko (Republic of Equatorial Guinea) seems to have cut malaria burden significantly [9]. These results suggest that aggressive malaria control is having a large impact on all-cause child mortality. The aggressive malaria control is totally dependent on full financial support, which may not be sustainable or durable. If funding is on a path of endless investment with little hope of sustainability, particularly the poor countries in SubSahara Africa, we can imagine what it would be that suppression of transmission for 5-10 years, and then loss of control, exposes a non-immune population to a severe epidemic.

Although the availability of effective malaria control tools is important, logistics for their full use in the integrated malaria control is a more perplexing issue [31]. Any delay or discontinuation of the integrated programme can make malaria return quickly. Too much emphasis on one of the measures is not helpful to eliminate malaria from this island. For example, overstating the effectiveness of IRS on malaria control can lead to the erroneous notion that yearly IRS is the only panacea which can induce 100% protection. It may take decades to eliminate malaria from this island even all the current measures are sustained [32]. Political commitment should reflect in the renewed attention to malaria interventions which are based on scientific evidence to develop long lasting control policies.

## Conclusions

The systematical deployment of IRS, LLINs, IPT, and ACT has made malaria become a small problem but any delay or interruption of these programmes may change the epidemiology of malaria from stable endemics to unstable epidemics in this island. Serious obstacles in the malaria control remain, which include poor performance of health care, inadequate use of LLINs, and lack of an effective malaria surveillance system. To avoid the waste of foreign aid, it is important to coordinate well all international participants and work in close cooperation. Strengthening of active surveillance with close follow-up as well as epidemic preparedness and response should the current priorities. All strategies currently applied should be enhanced and promoted equally to avert a possible large-scale epidemic in a low and unstable malaria transmission setting.

## Competing interests

The authors declare that they have no competing interests.

## Authors' contributions

PWL was responsible for the field work, study coordination, laboratory analysis including PCR confirmation, and data collection. CTL carried out the mosquito cultivation, bioassay, and supervised the field work. VEdR designed PCR analysis for evaluation and monitoring and helped in reviewing the manuscript and discussion. BdS took full responsibility for the integrity of the data and accuracy of the data and its analysis. HSR contributed to study coordination, and organized field work. MFS led the conceptual design, study coordination, supervision, data interpretation and manuscript preparation. All authors read and approved the final manuscript.

## Supplementary Material

Additional file 1 Associated indicators with malaria epidemics in São Tomé 2008 - 2009 Click here for file
