# Supplementary material for: Potential threat of malaria epidemics in a low transmission area, as exemplified by São Tomé and Príncipe
Source: Malar J. 2010 Sep 29;9:264. doi: 10.1186/1475-2875-9-264 (PMC2955676; doi:10.1186/1475-2875-9-264)
Supplement: Additional file 1 — Associated indicators with malaria epidemics in São Tomé 2008 - 2009 [file 1475-2875-9-264-S1.PDF]

Additional file 1. Associated indicators with malaria epidemics in São Tomé 2008 - 2009

District	Agua Grande	Me-Zoxi	Lobata	Cantagalo	Lemba	Caue
Population and density 2008	58661 (3555/km^2)	41174 (338/km^2)	17949 (171/km^2)	15256 (128/km^2)	12215 (53/km^2)	6583 (25/km^2)
3rd IRS finished in 2007 (coverage)	November (70%)	July (75%)	March (75%)	April (76%)	January (85%)	March (92%)
Prevalence Apr 2008	3. 5%	3.7%	4.1%	3.2%	2.0%	1.9%
Prevalence Apr 2009	4.3%	4.1%	4.2%	3.5%	ND	ND
Incidence 2008	2.1%	2.0%	2.3%	1.4%	0.9%	0.7%
Incidence 2009	3.9%	4.1%	3.5%	4.7%	0.8%	0.8%
Asymptomatic cure rate in Apr 2008 (%)	493/1037 (48%)	256/735 (35%)	327/647 (51%)	101/213 (47%)	142/157 (90%)	136/143 (95%)
Symptomatic cure rate in Jan – Jun 2009 (%)	1393/1536 (91%)	775/906 (86%)	612/659 (93%)	150/166 (90%)	100/107 (93%)	101/105 (96%)
Malaria epidemics	Nov 2008 – Jun 2009	Dec 2008 – Jun 2009	May – Jun 2008	Jan – Jun 2009	Nil	Nil
Months from last IRS to epidemics	12	16	14	20	-	-
